# Ensuring a high response rate to patient reported outcomes in surgical trials using incentives - a trial manager perspective

**DOI:** 10.1186/1745-6215-14-S1-O83

**Published:** 2013-11-29

**Authors:** Hanne Bruhn, Alison McDonald, Angus Watson, John Norrie

**Affiliations:** 1 University of Aberdeen, Aberdeen, UK; 2 NHS Highland, Inverness, UK

## 

Achieving a high response rate for patient reported outcomes (PROs) during follow-up in RCTs is challenging. A response rate of 69% to the 12 month questionnaire in the first 20% of participants reaching the timepoint in eTHoS triggered the team to intervene. eTHoS is an ongoing surgical trial for haemorrhoidal disease with the primary outcome point at 24 months and recruitment target of 800. Responses are routinely collected by postal questionnaire or online so other methods to protect the outcome at 24 months had to be considered.

The literature suggests that a small voucher payment can increase the response rate although the evidence for the effectiveness in large RCTs is fairly limited. We have designed a sub-study to investigate randomly allocating participants to receive a £5 voucher with their questionnaire at 12 and/or 24 months.

In terms of the trial management there were several ethical and practical considerations.

Ethical considerations included fairness in distribution of the vouchers; whether to make the voucher conditional or unconditional and being inclusive in the kind of voucher to source. When using vouchers the sponsor also has several requirements that have to be fulfilled which affect trial management including keeping track of participants that have been sent a voucher and a requirement that participants be made aware that, depending on personal circumstances, the payment may be subject to tax.

The sub-study has received ethical approval and is awaiting R&D approval. Preliminary results and challenges in set-up will be presented and discussed.

